# Prognostic values of YTHDF1 regulated negatively by mir‐3436 in Glioma

**DOI:** 10.1111/jcmm.15382

**Published:** 2020-05-25

**Authors:** Chenyang Xu, Bingjian Yuan, Tao He, Bingqian Ding, Song Li

**Affiliations:** ^1^ Henan University Kaifeng Henan P.R. China; ^2^ Department of Neurosurgery Huaihe Hospital of Henan University Kaifeng Henan P.R. China; ^3^ Department of Urology Huaihe Hospital of Henan University Kaifeng Henan P.R. China

**Keywords:** glioma, miRNA, prognostic signature, RNA modification, YTHDF1

## Abstract

M6A methylation is likely to be closely associated with the occurrence and development of tumours. In this study, we demonstrated that the transcription levels of the m6A RNA methylation regulators are closely related to the prognosis of glioma. Univariate Cox analysis was performed on the expression levels of methylation regulators and selected three hub genes in glioma. Next, we systematically compared the expression of these m6A RNA methylation regulators in gliomas with different clinicopathological features. The overall survival (OS) curve of the hub genes was initially established based on TCGA database information. YTHDF1 was selected from the hub genes following survival and prognosis analysis. A nomogram was developed to predict the survival probability. We further performed cell function and in vivo xenograft tumour experiments to further verify its role in tumour progression. Next, based on the miRanda and miRDB databases, we predicted one microRNA, hsa‐mir‐346, that might regulate and bind to 3'UTR of YTHDF1, which was confirmed by our fluorescent enzyme reporter gene experiment. In summary, m6A RNA methylation regulators play a potential role in the progression of gliomas. YTHDF1 may have an essential function in glioma diagnosis, treatment and prognosis.

## 1 INTRODUCTION

Glioma is a common primary central nervous system tumour. Approximately 200,000 new patients are diagnosed every year around the world. The most frequent type of malignant glioma (WHO III–IV) is glioblastoma multiforme (GBM), which accounts for 45.2% of all central nervous system malignancies, with a median overall survival of only 12‐18 months.^1^ The treatment of glioma has evolved from a single surgical treatment to a combined treatment such as surgery plus radiotherapy and chemotherapy, but the prognosis has not improved significantly. At present, the clinical diagnosis of glioma includes imaging and histopathology that also have limitations. Therefore, it is important to reveal the mechanisms of glioma treatment and prognosis to find new diagnostic targets.

N6‐adenine (m6A) is the most abundant chemical modification in eukaryotes, and m6A methylation is an epigenetic modification of RNA molecules. In the 1970s, geneticists discovered this methylation modification on eukaryotic messenger RNA (message RNA, mRNA).^2^ The function and role of m6A methylation in different biological processes have again attracted attention. A large number of studies have confirmed that with mainly three types of proteins are involved in m6A methylation. The first is m6A methyltransferase, whose coding gene is called Writer.^3^, ^4^, ^5^ The second type of protein is m6A demethylase, whose encoding gene is Erasers, which can remove m6A methylation groups in RNA.^6^ Their coding genes are called readers, including two subtypes of *YTHDFs* and *YTHDCs*.^7^, ^8^ *YTHDF1,* as a member of the m6A‐modified RNA‐binding protein family, inhibits its expression in normal lung epithelial cells to resist hypoxia‐induced apoptosis and is highly expressed in non‐small cell lung cancer tumour tissues and cell lines.^9^ Nishizawa et al. confirmed that YTHDF1 can affect tumour progression by modifying m6A methylation levels of inhibitory genes.^10^ Another analysis of clinical data showed that patients with m6A hypomethylation had significantly lower disease‐free survival (DFS) and overall survival (OS) and a higher recurrence rate (*P* < 0.01).^11^ However, there is a lack of literature on the function and prognostic value of regulators in the malignant progression of gliomas.

We systematically compared the expression of these m6A methylation genes in gliomas with different clinicopathological features of their TCGA datasets. *YTHDF1* was selected from the hub genes based on survival and prognosis analysis. Multivariate cox regression analyses were performed, and a nomogram was built with potential risk factors based on a multivariate Cox analysis to predict survival probability. We further performed cell function and in vivo xenograft tumour experiments to further verify its role in tumour progression. Next, based on the miRanda and miRDB databases, we predicted one microRNA, *hsa‐mir‐346*, that might regulate and bind to 3'UTR of *YTHDF1*, which was confirmed by a fluorescent enzyme reporter gene experiment.

## 2 MATERIALS AND METHODS

### 2.1 Microarray data

The gene expression profiles and clinical information were downloaded from The Cancer Genome Atlas (TCGA). We selected the expression data of thirteen m6A RNA methylation regulator‐related gene from the TCGA databases and then compared the associations between different clinical parameters and m6A RNA methylation regulator expression in gliomas. The differential expression of any gene of interest between tumour and adjacent normal tissues was studied in gliomas using R software (version 3.5.1). Analyses of the differential expression and the pairwise difference map of these m6A RNA methylation regulators were performed using R software.

### 2.2 Kaplan‐Meier analysis

We compared the levels of genes expression at the clinical stage using R software. The overall survival analyses of the hub genes were performed trough TCGA databases. Kaplan‐Meier plots and mutation of hub genes were analysed by the open‐source web tool cBioPortal (http://www.cbioportal.org/index.do).

### 2.3 Prognostic nomogram

Multivariate analyses were conducted to determine the prognostic value of the hub genes and clinical characteristics. A nomogram and risk score were built with potential risk factors (*P* < 0.05) based on a multivariate Cox analysis using the R software package. The predictive performance of the nomogram was then measured by receiver operating characteristic (ROC) curves and analysed by Kaplan survival probability.

### 2.4 Cell culture and transfection

Glioma cell lines were cultured in DMEM medium containing 10% foetal bovine serum under the conditions of 37°C, 5% CO_2_ and 100% saturated humidity. Passive culture was performed when the cells confluent to 70% to 80%. The cells were cultured in 6‐well plates and synchronized with serum‐free medium for 24 hours. Further, the cells were divided into a Si‐NC group and a Si‐*YTHDF1* group. Next, we transfected the blank plasmid and *YTHDF1* interfering plasmid following the instructions of the Lipofectamine 2000 transfection kit (Guangzhou Xiangbo Biotechnology Co., Ltd., Guangzhou, China).

### 2.5 Quantitative real‐time polymerase chain reaction and Western blotting

Qqt‐PCR was used to detect the changes in the *YTHDF1* expression. The aforementioned reverse transcription products were tested by Takara's SYBRPremixExTaqTM on an ABI7900 instrument using qPCR, and GAPDH expression was utilized as an internal reference. The following primers were used: *YTHDF1*‐5′ACCTGTCCAGCTATTACCCG, TGGTGAGGTATGGAATCGGAG; GAPDH‐5′ACCCACTCCT CCACCTTTGA, 3′CTGTTGCTGTAGCCAAATTCGT. Cells from each group were collected, and total cell protein was extracted using cell lysate. Then, the protein concentration was determined by a BCA kit. According to the results of protein concentration detection, 25 μL of protein samples was subjected to Western blot detection.

### 2.6 Colony formation assay

We diluted the cell suspension of a single‐layer culture cell in logarithmic growth phase by multiple gradients and inoculated the culture dish with an appropriate cell density (according to the proliferation ability). Next, we discarded the supernatant and carefully dipped it twice with PBS, added 5 mL of pure methanol or 1:3 acetic acid/methanol and fixed it for 15 minutes. Then, we removed the fixative solution, added an appropriate amount of Giemsa stain and applied the staining solution for 10‐30 minutes. Finally, we inverted the plate, superimposed a grid of transparencies and counted the clones directly with the naked eye.

### 2.7 In vivo xenograft tumour growth

Twelve nude mice were divided into an experimental and a control group (n = 6). Nude mice were kept in an environment of about 25°C with a suitable humidity and were provided with sufficient food and water during the administration. Experimental grouping: About 5 × 106 cells transfected with pc‐*YTHDF1* in SHG‐44 cell (si‐*YTHDF1* in U87 cell) were suspended in 0.1 mL of PBS and injected subcutaneously into nude mice. The control group was given the same amount of normal saline. The volume and weight of the xenograft tumours in the nude mice were then measured every 10 days. Thirty days later, the nude mice were killed, and the transplanted tumours were removed. The morphological changes in the tumour tissues were observed by H & E staining, and the expression of Ki67 was also detected.

### 2.8 Immunohistochemical analysis

Antibody system immunohistochemistry kits were purchased from Roche, Switzerland. According to the method of Dowset et al,^12^ pale yellow, yellow or brown particles appeared in the nuclei of cells as positive expression. Readers blinded to the patient's pathological data observed the expression of the whole film under a microscope.

### 2.9 miRNA database analysis

The potential miRNAs targeting *YTHDF1* were downloaded from miRanda and miRDB databases. We performed survival analysis of *hsa‐miR‐346* in brain glioma, downloaded from TCGA. We then conducted survival analysis of *hsa‐miR‐346* in glioma using a TCGA project. Coexpression analysis for *hsa‐miR‐346* and *YTHDF1* was performed via ENCORI, which mainly focuses on miRNA‐target interactions and is an open‐source platform for studying RNA‐RNA interactome data (http://starbase.sysu.edu.cn/index.php).

### 2.10 Luciferase reporter assay

The *YTHDF1* gene promoter was cloned by RT‐q PCR and DNA fragments from the 3′‐UTR of *YTHDF1* inserted into the luciferase reporter vector pGL3. Then, the pGL3‐*YTHDF1*‐3′UTR reporter plasmids (100 ng) plus 5 ng of pRL‐TK renilla plasmid and increasing levels of NC, *Hsa‐mir‐346* or mutant *Hsa‐mir‐346* mimics were co‐transfected into the U87 cell. Luciferase activity analysis was next performed to calculate the luciferase activity ratio of the reporter plasmid and the internal reference.

### 2.11 Statistical analysis

Statistical analysis was conducted using SPSS (IBM, Chicago, IL, USA) and R software (version 3.5.1). Factors were identified as significant at *P* < 0.1 in the univariate analysis. Comparisons between groups were performed using independent sample t tests, and multiple group comparisons were done using single‐factor variance. Pairwise comparisons were performed using LSD Lt test. *P* < 0.05 was considered statistically significant.

## 3 RESULTS

### 3.1 Clinical significance of *YTHDF1*

**Figure 1:**
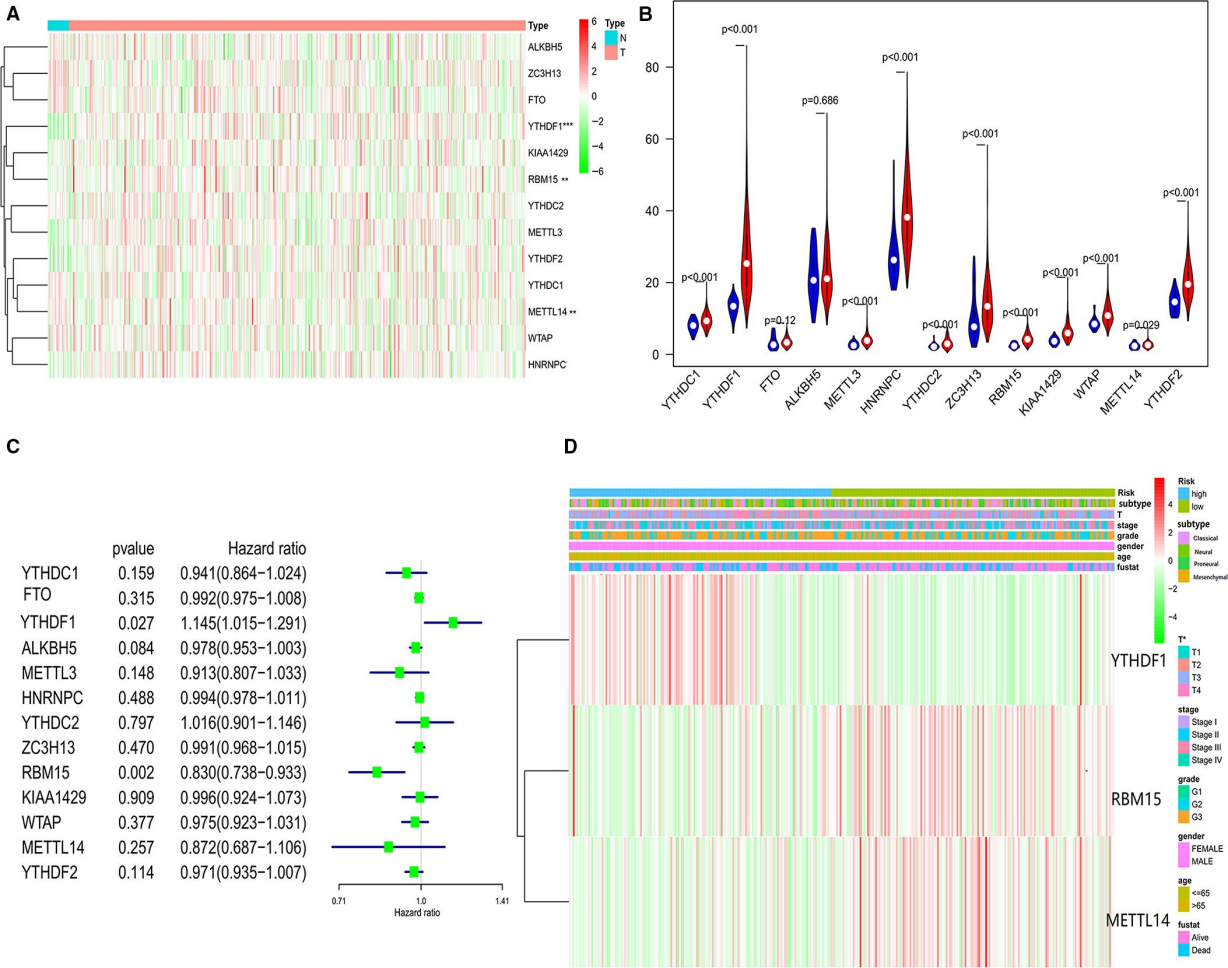
Heat maps (A) and violin (B) plot showed different gene expression profiles in the normal vs tumour group in glioma from TCGA. C, A univariate Cox regression analysis on the expression levels of thirteen genes in the TCGA dataset. D, Heat maps showed that the different gene expressions of YTHDF1, METTL3 and FTO are significantly correlated with clinicopathological features. *P < 0.05 versus control

We examined the microarray data of all 605 glioma cases from the TCGA database. Heat maps and violin plot showed different gene expression between the normal and the tumour group (Figure 1A,B). As is visible in the violin plot, the mRNA expression levels of Y*THDF2, YTHDF1, METTL3, RBM15* and *HNRNPC* were up‐regulated in glioma compared to normal tissues, whereas the expression levels of *ALKBH5, WTAP, YTHDC2, ZC3H13* and *METTL14*, especially of *FTO*, were decreased in glioma (Figure 1B). Next, we performed univariate cox regression analysis on the expression levels in the TCGA dataset (Figure 1C). As can be seen in Figure 1D, *YTHDF1*, *RBM15* and *METTL14* are associated with clinicopathological features (*P* < 0.05; Figure 1D). Of these genes, *YTHDF1* is the risky gene with HR > 1, whereas *RBM15* and *METTL14* are protective genes.

**Figure 2:**
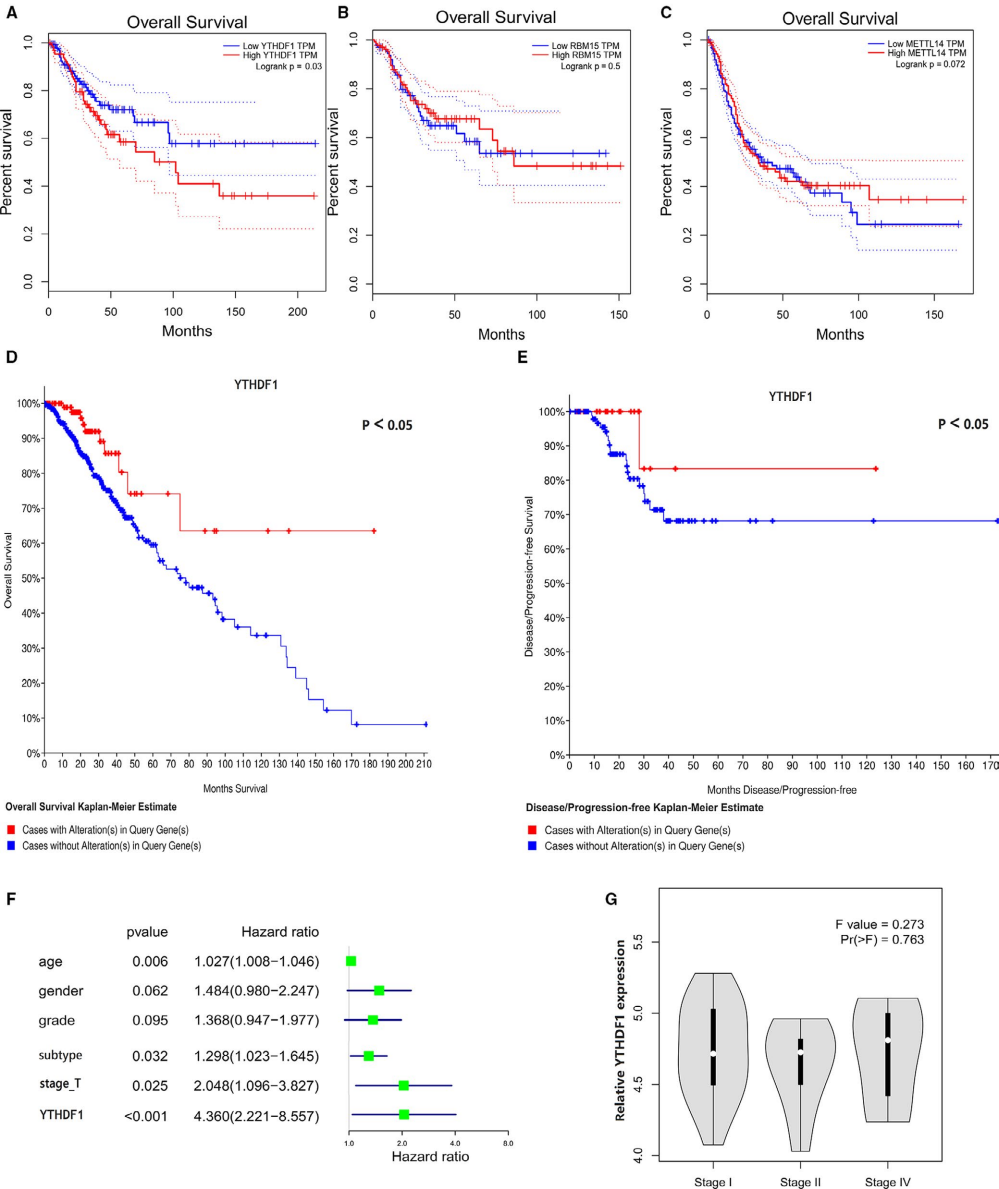
A‐C, The overall survival (OS) curve of YTHDF1, METTL3 and FTO was performed firstly on Kaplan‐Meier Plotter Database. The overall survival (D) and disease‐free (E) survival of the YTHDF1 mutations on glioma were performed via the cBioPortal for Cancer Genomics website. F, The high expression levels of YTHDF1 are correlated with advanced stages. G, The multivariate analysis of dimension, subtype, age, grade, stage and YTHDF1 were statistically significant factors for glioma progression. *P < 0.05 versus control

To evaluate the prognosis of the three tested genes in glioma, the overall survival (OS) curve was performed first on the Kaplan‐Meier Plotter Database. The high expression of *YTHDF1* in glioma was associated with worse OS (*P* < 0.05; Figure 2A). OS of *RBM15* and *METTL14* were not statistically significant (Figure 2B,C). Interestingly, the OS and DFS of the *YTHDF1* mutations in glioma were performed via the cBioPortal. The results we obtained showed that glioma cases with related genes mutations had better OS and DFS (*P* < 0.05; Figure 2D,E). Next, we investigated the association between the *YTHDF1* gene expression and the clinical stage using the TCGA database. The results indicated that high levels of *YTHDF1* are correlated with advanced stages. The above results indicated that *YTHDF1* may contribute to glioma progression (Figure 2F,G).

### 3.2 Prognosis of *YTHDF1* in glioma patients

**Figure 3:**
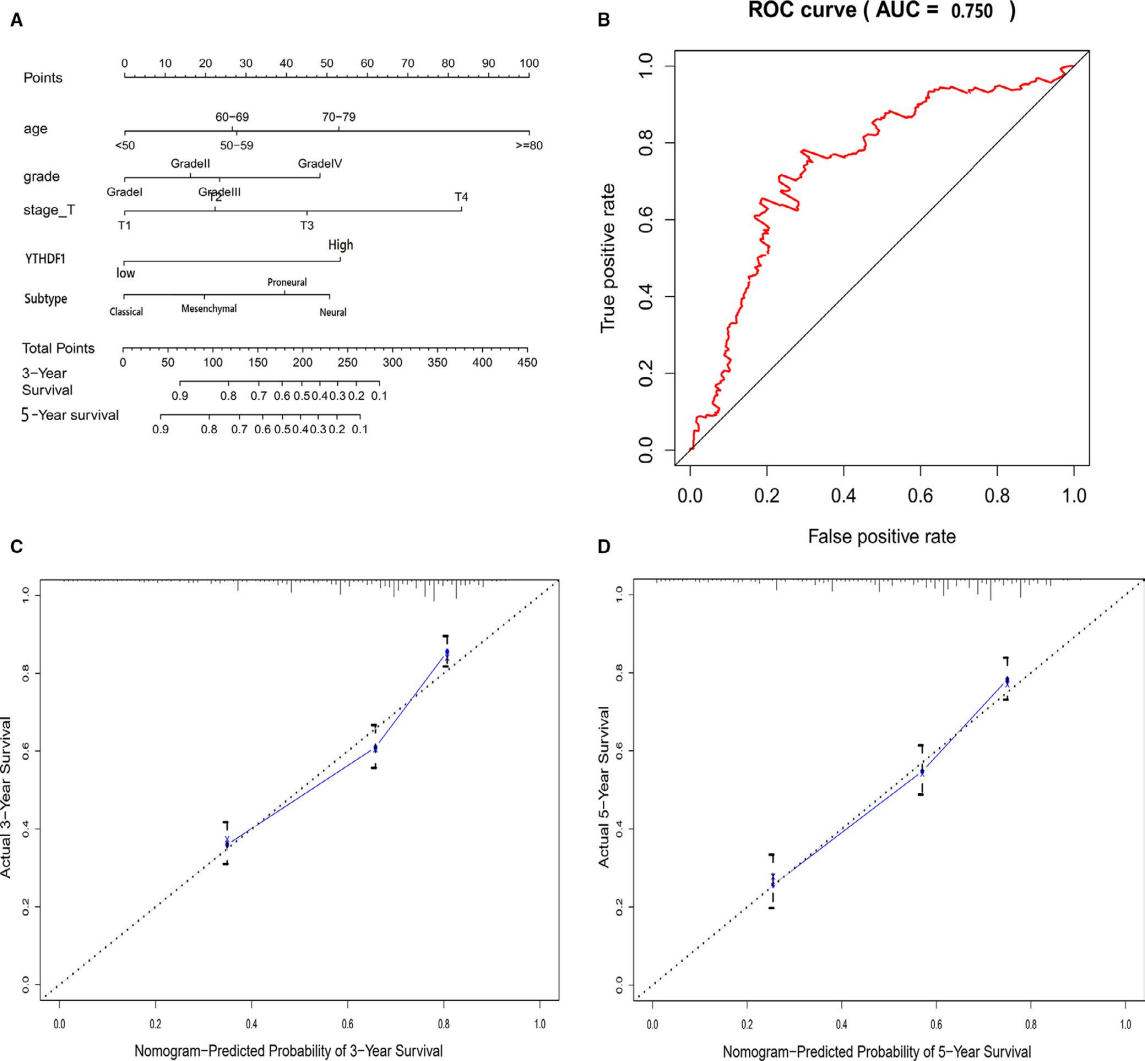
A, The prognostic nomogram that integrated all significant independent factors from the multivariate analysis for OS in the training cohort. B, The ROC curve area evaluating the prognostic nomogram for OS prediction was 0.750 (95% CI, 0.729 to 0.759). C, The calibration plot for the probability of survival at 3 or 5 years after surgery showed an optimal agreement between the prediction by the nomogram, respectively. *P < 0.05 versus control

In the present study, a total number of 605 glioma patients were included from TCGA. The clinicopathological characteristics of the patients are listed in Table 1. The results of the multivariate analysis revealed that the dimension, subtype, age, grade, stage and *YTHDF1* were statistically significant factors for glioma progression (Figure 2F). The prognostic nomogram that integrated all significant independent factors is presented in Figure 3A. The ROC curve area evaluating the prognostic nomogram for OS prediction was 0.750 (95% CI, 0.729 to 0.759; Figure 3B). The calibration plot for the probability of survival at three or five years revealed an optimal agreement with the prediction by the nomogram, which appeared to be good in the model (Figure 3C,D).

**Table 1:** Demographic and clinicopathological characteristics of patients with Glioma in TCGA database

Demographic or characteristics	All patients (N = 605) (%)	Training cohort (N = 300)	Validation cohort (N = 305)
Age at diagnosis			
≤49	69 (11.4)	36 (11.6)	37 (11.8)
50‐59	152 (25.1)	73 (24.3)	78 (25.7)
60‐69	145 (24.0)	73 (24.3)	72 (23.9)
70‐79	132 (21.8)	69 (22.6)	68 (23.6)
≥80	107 (17.7)	49 (16.6)	49 (16.0)
Gender			
Female	241 (39.8)	241 (39.8)	241 (39.8)
Male	364 (60.2)	364 (60.2)	364 (60.2)
Subtype			
Classical	156 (28.5)	84 (28.0)	87 (28.5)
Neural	100 (16.5)	51 (17.0)	50 (16.5)
Proneural	155 (25.6)	78 (26.0)	78 (25.6)
Mesenchymal	184 (30.4)	90 (30.0)	93 (30.4)
Grade			
G1	333 (55.0)	162 (54.0)	167 (55.0)
G2	200 (33.0)	102 (34.0)	99 (33.0)
G3	72 (12.0)	36 (12.0)	39 (12.0)
T (cm)			
0‐1	330 (54.6)	164 (54.7)	166 (54.4)
1‐1.4	196 (32.4)	99 (32.9)	97 (33.6)
1.5‐1.9	41 (6.9)	20 (6.6)	21 (6.8)
2‐3	35 (5.9)	16 (5.4)	19 (5.2)
Immunotherapy			
No	489 (80.9)	240 (80.0)	249 (80.2)
Yes	116 (19.1)	60 (20.0)	56 (19.8)
Radiation therapy			
No	426 (70.5)	212 (71.0)	214 (70.2)
Yes	179 (29.5)	87 (29.0)	92 (29.8)
YTHDF1 expression			
High	395 (65.4)	195 (65.0)	200 (66.0)
Low	210 (34.6)	105 (35.0)	105 (34. 0)

### 3.3 *YTHDF1* promotes the proliferation of glioma cell lines

**Figure 4:**
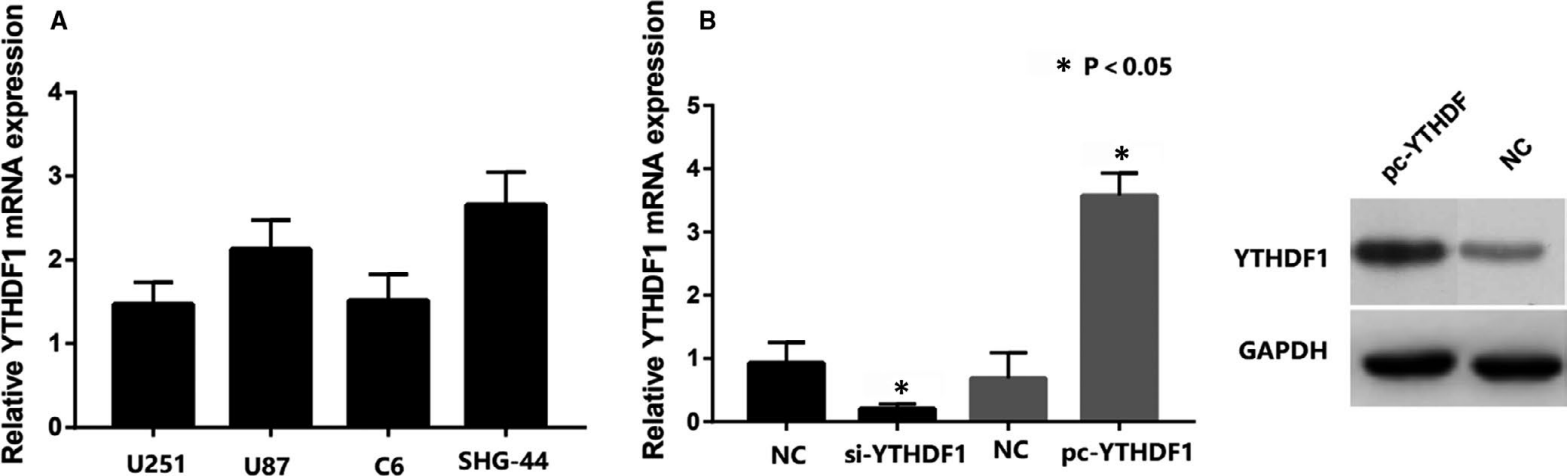
A, The YTHDF1 expression in different glioma cells. B, The expression efficiency of pc‐YTHDF1 and si‐YTHDF1 was measured by RT‐qPCR and WB assay in U87 and SHG‐44. *P < 0.05 versus control

**Figure 5:**
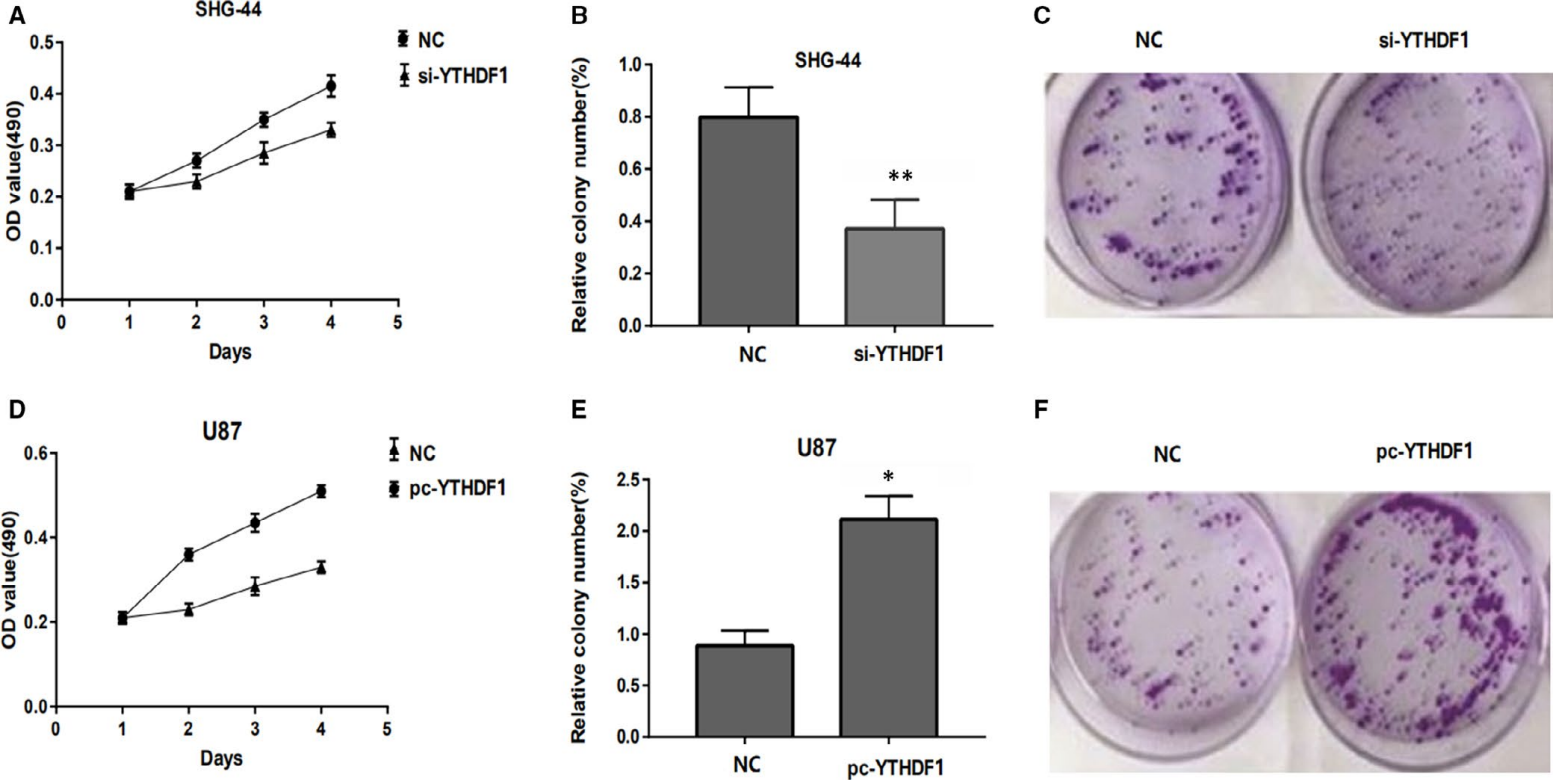
A, MTT assays show that YTHDF1 down‐regulation decreased cell proliferation in U87 shRNA cells. B, C, Colony formation assays indicate significantly decreased the number of colonies in the U87 shRNA cells compared to control U87 cells. D, MTT assays show that YTHDF1 overexpression increased cell proliferation in SHG‐44 shRNA cells. E, F, Colony formation assays indicate significantly decreased the number of colonies in the SHG‐44 shRNA cells compared to control SHG‐44 cells

First, RT‐qPCR assay was performed to investigate the expression of *YTHDF1* in different glioma cells. The results showed that the *YTHDF1* expression was markedly up‐regulated in U87 and SHG‐44 cell lines (Figure 4A). Then, the expression efficiency of pc‐Y*THDF1* and si‐*YTHDF1* was measured by RT‐qPCR assay in U87 and SHG‐44. Next, we overexpressed *YTHDF1* by transfecting a *YTHDF1* plasmid into a SHG‐44 cell line; a shRNA *YTHDF1* plasmid was transfected with a U87 cell line to knockdown the *YTHDF1* expression, indicating that introduction of si‐*YTHDF1* and pc‐*YTHDF1* is successful and can be used for subsequent research (Figure 4B). Then, the effects of *YTHDF1* down‐regulation and up‐regulation on cell proliferation and apoptosis were further examined in U87 and SHG‐44 cells, respectively (Figure 5A–F). MTS analysis showed that the proliferation ability of *YTHDF1* knockdown U87 cells was significantly lower than that of mock cells, and the proliferation ability of SHG‐44 cells with *YTHDF1* overexpression was significantly higher than that of mock cells (Figure 5A,D). Colony formation analysis showed that *YTHDF1* knockdown caused a significant decrease in the number of colonies in U87 cells, and *YTHDF1* overexpression caused a significant increase in the number of colonies in SHG‐44 cells (Figure 5B‐F).

### 3.4 *YTHDF1* promotes glioma tumour growth in vivo

**Figure 6:**
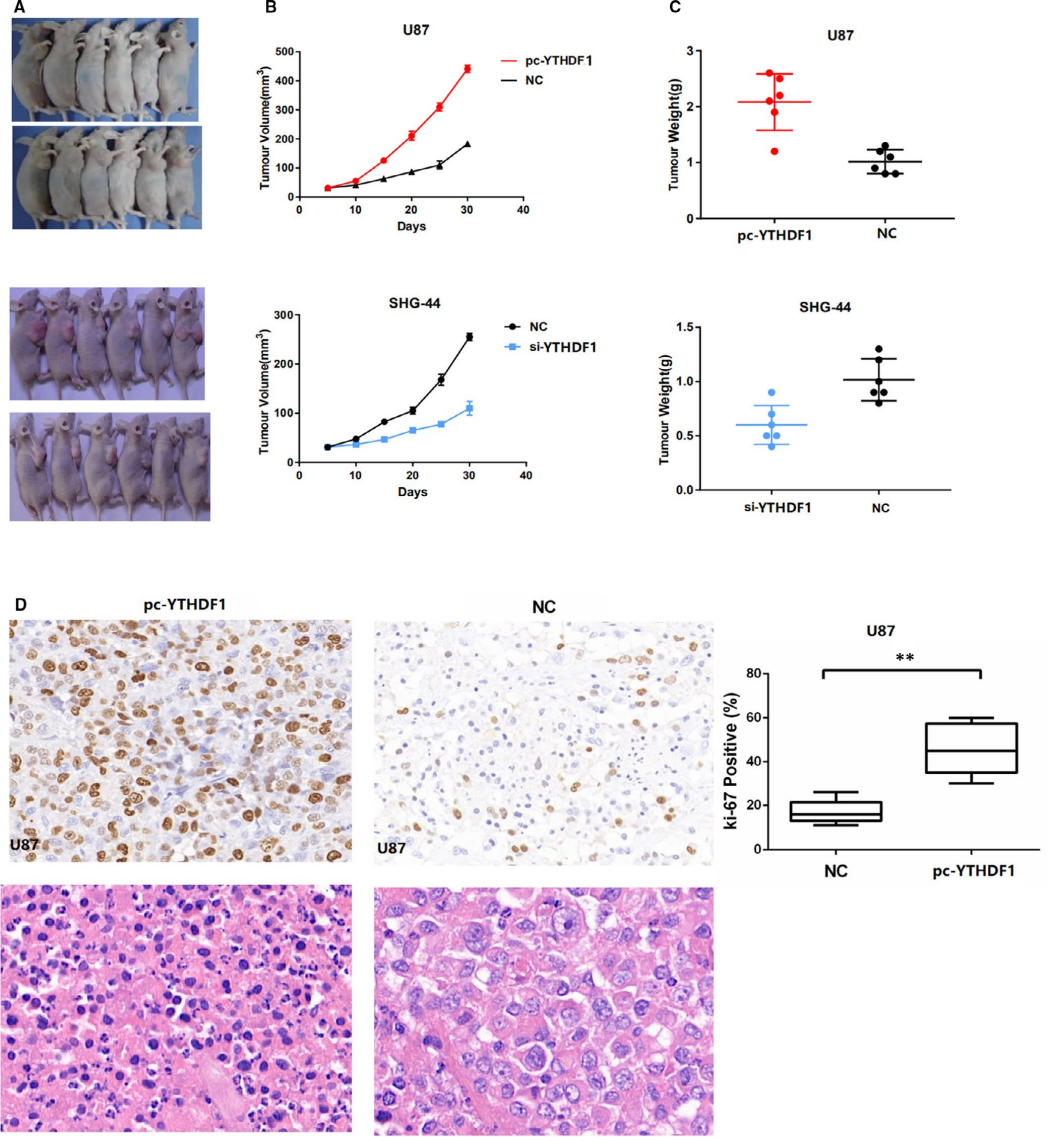
YTHDF1 promotes glioma tumour growth in vivo. A, Representative images of day 28 tumours in mice transplanted with U87‐shYTHDF1, U87 (control), SHG‐44‐YTHDF1 and SHG‐44 (control) cells. B, Plots showing tumour growth measurements of U87‐shYTHDF1, U87 (control), SHG‐44‐YTHDF1 and SHG‐44 (control) cells is shown. C, The mean tumour weights in each group (U87‐shYTHDF1, U87 (control), SHG‐44‐YTHDF1 and SHG‐44 (control) cells) on day 30 are shown. D, IHC staining showing that cell proliferation (Ki67‐positive) positively correlates with YTHDF1 expression levels. Data in (B) and (C) are presented as mean ± SD (n = 5). *P < 0.05 versus control

To further study the biological significance of *YTHDF1* in gliomas, we injected U87‐si*YTHDF1* and SHG‐44‐pc*YTHDF1* cells and their corresponding controls subcutaneously in nude mice and monitored their tumour growth. Compared to the control U87 cells, the U87‐pc*YTHDF1* cells had a significantly larger tumour size. In contrast, the SHG‐44‐si*YTHDF1* cells formed tumours that were much smaller than those in the SHG‐44 control cells (Figure 6A,B). In addition, the low *YTHDF1* levels in glioma cells were associated with low tumour growth rates and smaller tumour weight, whereas the high *YTHDF1* levels in glioma cells were associated with higher tumour growth rates and tumour weight (Figure 6C). Immunohistochemical experiments established that the Ki67 positive rate in the high‐*YTHDF1* group was significantly higher than that in the control group (Figure 6D). Taken together, these data suggest that *YTHDF1* promotes the growth of glioma tumours in vivo.

### 3.5 *Hsa‐mir‐346* targets *YTHDF1*

**Figure 7:**
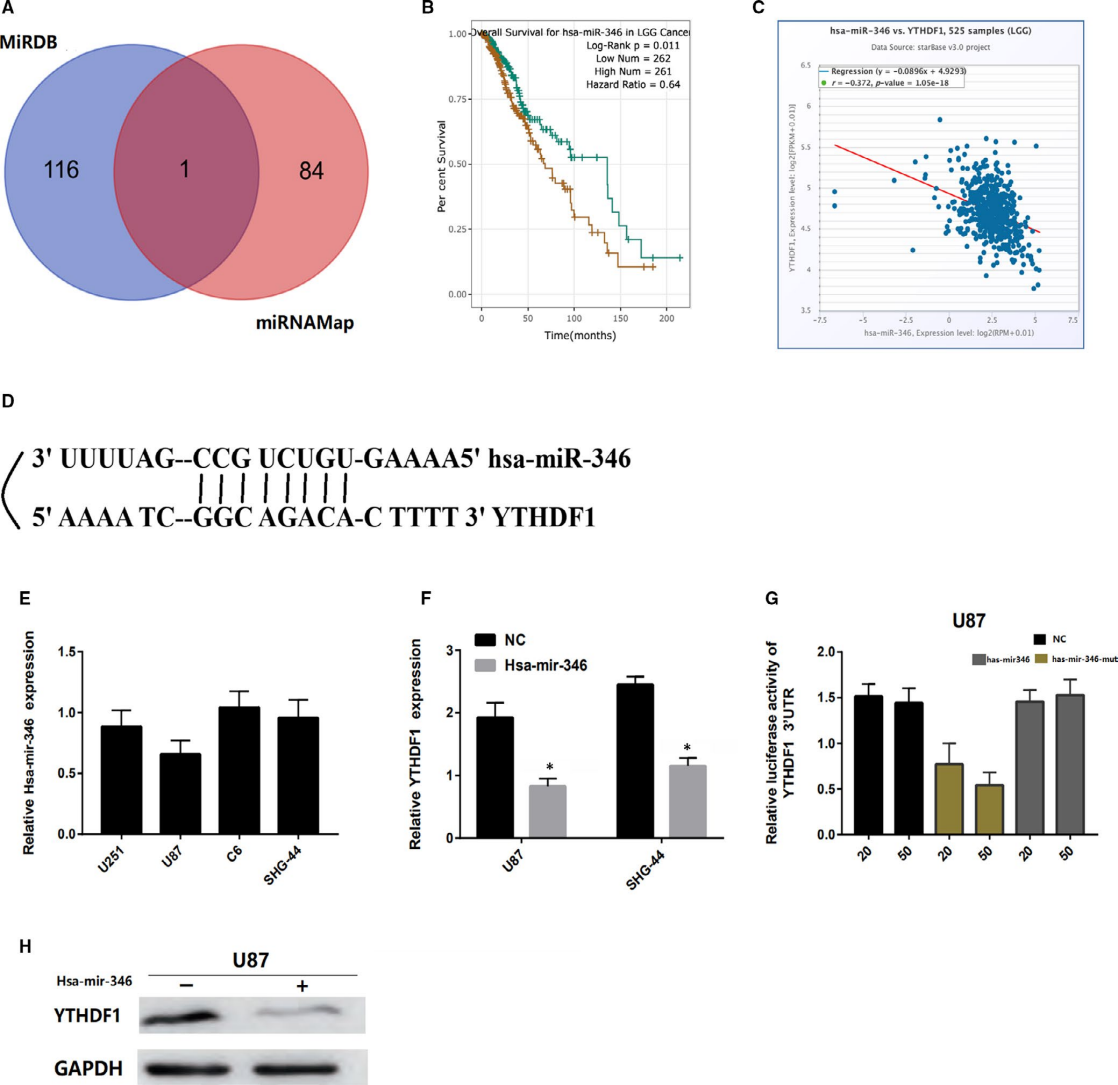
YTHDF1 is a target for miR‐346 in glioma cells. A, Venn diagrams showing the number of potential miRNAs targeting the 3'UTR of YTHDF1, as predicted by two databases, miRanda and miRDB. B, Survival analysis of hsa‐miR‐346 in brain glioma via ENCORI. C, The coexpression between hsa‐mir‐346 and YTHDF1 via ENCORI showed that hsa‐mir‐346 expression is negatively correlated with YTHDF1 expression. D, Sequences of miR‐346 and their potential binding sites in the 3'UTR of YTHDF1 are shown. E, Quantitative real‐time PCR analysing miR‐346 expression relative to U87 as internal control is shown. F, H Comparison of YTHDF1 expression in glioma cells transfected with miR‐346 mimic or negative control (NC) based on qRT‐PCR and Western blotting. The loading control for Western blotting was GADPH. G, Analysis of luciferase activity from reporters containing the 3'UTR end of YTHDF1 in cells transfected with the miR‐346 mimic, miR‐346 mutation mimic (miR‐346‐mut) and negative control (NC) is shown. *P < 0.05 versus control

Finally, we selected two sets of miRNAs related to *YTHDF1* from miRanda and miRDB databases and obtained *miR‐486‐5p* as a possible candidate by drawing Veen map (Figure 7A). Next, we performed survival analysis of *hsa‐miR‐346* in brain glioma via ENCORI. We found that glioma patients with high expression of *Hsa‐mir‐346* had better overall survival than those with low expression (*P* < 0.05; Figure 7B). Moreover, the miRNA‐target coexpression between *hsa‐mir‐346* and *YTHDF1* via ENCORI showed that *hsa‐mir‐346* expression was negatively correlated with the *YTHDF1* expression (Figure 7C). Importantly, we established that hsa‐mir‐346 may be combined with 3′UTR of *YTHDF1* (Figure 7D). Therefore, we speculated that *hsa‐mir‐346* is a potential key upstream negative regulator of *YTHDF1* and may be involved cancer treatment and prognosis.

In addition, we speculated that *hsa‐miR‐346* is a potential key upstream negative regulator of *YTHDF1* that may be associated with cancer treatment. Next, we analysed the expression level of *hsa‐mir‐346* in glioma cell lines by qRT‐PCR. The results showed that *hsa‐mir‐346* was down‐regulated in glioma cells (Figure 7E,F). Then, we transfected glioma cells or mutant *hsa‐mir‐346* mimics and luciferase‐labelled NEK2‐3'UTR with *hsa‐mir‐346*, which were subjected to luciferase reporter gene analysis. We found that *hsa‐mir‐346* mimic significantly reduced the activity of *YTHDF1*‐3'UTR, whereas the mutant *hsa‐mir‐346* mimic did not inhibit the luciferase activity of *YTHDF1*‐3'UTR (Figure 7G). Moreover, we analysed the expression levels of *YTHDF1* in u87 cells transfected with *hsa‐mir‐346* and the negative control (NC) by qRT‐PCR and Western blotting and found that the expression of YTHDF1 with *hsa‐mir‐346* was significantly lower than that of the negative control group (Figure 7H). Therefore, these results indicated that *YTHDF1* is regulated by *hsa‐mir‐346* and may have an impact on the prognosis of glioma patients.

## 4 DISCUSSION

Previous studies have reported that the pathological grade, age at onset, surgical methods and post‐operative adjuvant treatment of glioma patients are directly related to their prognosis.^13^, ^14^ However, many factors affect the prognosis of glioma patients, most of which are uncertain.^15^ Furthermore, much of the literature on the relationship between M6A methylation and tumours has been mostly focused on liver and cervical cancer. For example, Dominissini confirmed that the knockout of *YTHDF1* in cell lines induced apoptosis of liver cancer cells in in vitro experiments and speculated that its mechanism may be through the activation of the p53 signalling pathway.^16^ JmzhaoMa found that the expression of *YTHDF1*in liver cancer was reduced, which was negatively correlated with the prognosis of liver cancer patients.^17^ Patients with m6A hypomethylated cervical cancer had significantly lower disease‐free survival (DFS) and overall survival (OS), whereas hypomethylation led to a higher recurrence rate (*P* < 0.01).^11^ In this study, we demonstrated that the expression of regulators (RNA m6A RNA methylation) is also closely associated with the progress and prognosis of gliomas. Among them, *YTHDF1 is* associated with OS and clinicopathological features (*P* < 0.05). Importantly, *YTHDF1* is a risky gene with HR > 1 (*P* < 0.05).

Nishizawa et al found that the *YTHDF1* gene was highly expressed in colorectal cancer patients and was related to the tumour diameter, clinical stage, but its specific regulatory mechanism was not studied.^19^ Additionally, Hao et al conducted research on liver cancer data in TCGA and found that *YTHDF1* was associated with tumour progression and may be an indicator factor for poor prognosis.^20^ The results of these studies are consistent with our findings that the high expression of *YTHDF1* in glioma is associated with worse OS and advanced stages. Furthermore, glioma patients with *YTHDF1* mutations have a better overall survival and disease‐free survival than those of the wild‐type. In our investigation, univariate analysis revealed that ages, grade, stage and *YTHDF1* expression were statistically significant factors for glioma progression. Then, a prognostic nomogram that integrated all significant independent factors from the multivariate analysis for OS was made. Yan et al developed a radiomic signature to predict OS for glioma, and the C‐Index for OS prediction was 0.707.^21^ Our nomogram has five readily available pathological variables and achieved a ROC curve area of 0.750, superior to that of the nomogram, which indicated that the high *YTHDF1* expression was an independent risk factor and improved the current prognostic model. We further performed cell function and in vivo xenograft tumour experiments to further verify its role in tumour progression.

MicroRNAs (miRNAs) are a wide range of regulatory non‐coding RNAs (18‐25 nt) in mammals that can regulate the expression of target genes through post‐transcriptional regulation. At present, abnormally expressed miRNAs are often used as the key to disease prediction, prevention and treatment in clinical treatment.^21^, ^22^, ^23^ Next, based on the data available in the miRanda and miRDB databases, we predicted that one microRNA, *hsa‐mir‐346*, might regulate and bind to 3'UTR of *YTHDF1*, which was later confirmed by a fluorescent enzyme reporter gene experiment. Previous studies have shown that hsa‐miR‐346 can regulate other physiological and pathological processes, including cell differentiation, inflammatory reactions and carcinogenesis.^24^, ^25^, ^26^

It is noteworthy that *miR‐346* was found to be up‐regulated in human cervical cancer tissues and promoted malignant progression.^28^ Here, using ENCORI, we found that glioma patients with high expression of *hsa‐mir‐346* had better overall survival than those with low expression (*P* < 0.05). Importantly, the miRNA‐target coexpression between *hsa‐mir‐346* and *YTHDF1* established by ENCORI showed that the *hsa‐mir‐346* expression was negatively correlated with the *YTHDF1* expression. Next, we confirmed that *hsa‐mir‐346* mimic significantly reduced the luciferase activity of *YTHDF1*‐3'UTR by luciferin experiments. There is evidence that the m6A reader *YTHDF1* can enhance the translation of *EIF3C* and its expression, promoting the malignant progression of ovarian cancer.^29^ Therefore, we speculated that *hsa‐miR‐346* is a potential key upstream negative regulator of *YTHDF1* that may be related to cancer treatment and prognosis. However, this notion needs to be further explored.

In summary, our study showed that *YTHDF1* is associated with glioma progression, and high *YTHDF1* expression also predicts a poor prognosis in glioma patients. We also developed a nomogram to predict the overall survival (OS) of glioma patients. Finally, we identified *hsa‐mir‐346* as an upstream regulator of *YTHDF1* expression, which may be involved in the development of a new treatment reducing the development of glioma.

## CONFLICT OF INTEREST

The authors declare that they have no conflicts of interest.

## AUTHOR CONTRIBUTION

SL and CX conceived and designed the study, which were proofed by SL. CX analysed the data and wrote the manuscript. BY and TH carried out the concepts, design and manuscript preparation. BY, TH and BD provided assistance for data acquisition. All authors have read and approved the content of the manuscript.

## Data Availability

The data that support the findings of this study are available from the corresponding author upon reasonable request.
